# Cwc24p Is a General *Saccharomyces cerevisiae* Splicing Factor Required for the Stable U2 snRNP Binding to Primary Transcripts

**DOI:** 10.1371/journal.pone.0045678

**Published:** 2012-09-24

**Authors:** Patricia P. Coltri, Carla C. Oliveira

**Affiliations:** Department of Biochemistry, Institute of Chemistry, University of São Paulo, São Paulo, SP, Brazil; NIGMS, NIH, United States of America

## Abstract

Splicing of primary transcripts is an essential process for the control of gene expression. Specific conserved sequences in premature transcripts are important to recruit the spliceosome machinery. The *Saccharomyces cerevisiae* catalytic spliceosome is composed of about 60 proteins and 5 snRNAs (U1, U2, U4/U6 and U5). Among these proteins, there are core components and regulatory factors, which might stabilize or facilitate splicing of specific substrates. Assembly of a catalytic complex depends on the dynamics of interactions between these proteins and RNAs. Cwc24p is an essential *S. cerevisiae* protein, originally identified as a component of the NTC complex, and later shown to affect splicing *in vivo*. In this work, we show that Cwc24p also affects splicing *in vitro*. We show that Cwc24p is important for the U2 snRNP binding to primary transcripts, co-migrates with spliceosomes, and that it interacts with Brr2p. Additionally, we show that Cwc24p is important for the stable binding of Prp19p to the spliceosome. We propose a model in which Cwc24p is required for stabilizing the U2 association with primary transcripts, and therefore, especially important for splicing of RNAs containing non-consensus branchpoint sequences.

## Introduction

Eukaryotic gene expression is regulated at different levels. Splicing excises introns from primary transcripts and ligates exons forming mature RNA molecules. Splicing is catalyzed by the spliceosome, a complex macromolecular machinery composed of 5 snRNAs (U1, U2, U4/U6 and U5) and more than 100 proteins [1], [2]. Spliceosome assembly at each intron is dynamic and involves several RNA-RNA, RNA-protein and protein-protein rearrangements, which are necessary for the catalysis of two successive transesterification reactions that lead to the release of a mature RNA in an ATP-dependent manner. In *Saccharomyces cerevisiae*, the catalytic spliceosome is composed of ∼60 proteins, most of which are conserved among eukaryotes [3].

In *S. cerevisiae*, spliceosome assembly is dependent on the exact recognition of consensus sequences in the exon-intron boundaries and within the intron [4]. After U1 snRNP binding to 5′ splice site, the U2 snRNP joins the complex and performs rearrangements within the primary transcript-spliceosome complex. Interactions of the U2 snRNA in complex A include base-pair formation between a U2 single-stranded region and the branchpoint site in the intron [5]. After the joining of the tri-snRNP to the complex, several rearrangements on U2 stem loops IIa and IIc lead to U2∶U6 pairing, an important event for catalytic activation of the spliceosome [6]. The major RNP rearrangements during assembly are dependent on the activity of eight RNA helicases among which are Brr2p, responsible for U4/U6 unwinding, and Prp24p, responsible for the annealing of these two snRNAs *in vivo* [7], [8]. The ATP-dependent helicases are also important checkpoints for spliceosome assembly, and recent work suggests that the removal of SF3b proteins from the branchpoint is triggered by Prp2p activity, and may be an important rearrangement leading to the nucleophilic attack of the 5′ exon [9], [10], [11]. The second splicing step and the mature RNA release are dependent on the association of the helicases Prp16p and Prp22p, respectively [12], [13].

The great majority of *S. cerevisiae* introns have consensus splicing sequences. However, a few yeast primary transcripts have non-consensus splice signals [14], which can indicate the involvement of additional regulatory factors responsible for the recognition of the sequences and stabilization of the splicing complexes on these primary transcripts. In fact, a few examples of non-consensus splice signals were shown to require specific factors to complete splicing. The use of non-consensus 5′ splice signals might be related to the recruitment of Hub1p and its connection to U1 snRNP [15]. Interestingly, fourteen introns in yeast have a non-consensus branchpoint with a G instead of U in the first position (Ares Lab Yeast Intron Database, http://intron.ucsc.edu/yeast4.1/). An important question arising from these observations is to understand how the cell ensures the efficient splicing of these primary transcripts.

During spliceosome assembly, the association of the **n**ine**t**een **c**omplex proteins (NTC), formed by Prp19p, Cef1p and at least nine other components, rearranges interactions between snRNAs U5, U6 and the primary transcript, and has an important role in the catalytic activation [16], [17]. Previous work showed that the NTC destabilizes the binding of Lsm proteins to U6 snRNA, thus exposing the U-tract of this snRNA to new interactions, in this case, with the intron [17], [18]. It has been proposed that some NTC components associate within the spliceosome rather than forming a separate sub-complex before spliceosome association [16], [17]. Proteins isolated in association with Cef1p, such as Cwc21p, Cwc22p and Cwc25p, were shown to be important for rearrangements prior to the catalytic activation [19], [20], [21], [22], [23]. Cwc24p was also isolated in the complex with Cef1p [21]. Further work showed that *in vivo* depletion of this conserved 30 kDa essential yeast protein caused pre-rRNA processing defects, leading to accumulation of the 35S pre-rRNA [24]. A closer inspection of splicing activity in a conditional strain depleted of Cwc24p showed reduced splicing efficiency. Specifically, splicing of the transcripts snR17A and B were strongly affected by the depletion of Cwc24p, leading to decreased levels of the mature snoRNA U3, and consequently causing pre-rRNA processing defects [24].

Despite the observations described above, the mechanism by which Cwc24p affects splicing remained to be determined. In this work, we show that the depletion of Cwc24p severely affected splicing *in vitro* in general, with a strong impact on the pre-U3 RNA. Interestingly, pre-U3 transcript has a non-canonical branchpoint site, which could require additional factors for efficient spliceosome assembly. We observed reduced levels of U2 snRNA in splicing complexes formed in the absence of Cwc24p, strongly suggesting a weaker association of this snRNA. These results suggest that Cwc24p is important for spliceosome assembly, especially on substrates with non-canonical branchpoint sequences such as pre-U3. *In vitro* analysis confirmed the Cwc24p requirement for splicing of pre-U3, pre-TEF4, and pre-ACT1, thus characterizing this protein as a general splicing factor, involved in the association of U2 snRNP with the spliceosome.

## Materials and Methods

### Yeast strains and media

*S. cerevisiae* strains used in this work (Table 1) were cultivated at 30°C in rich (YPD) or minimal (YNB) media supplemented with the required amino acids.

**Table 1:** *Saccharomyces cerevisiae* strains used in this work.

Strain	Genotype	Reference
BJ2168	MATa prc1 prb1 pep4 leu2 trp1 ura3	S.C.Cheng
W303/GAL-A	MATa/MATα ura3-52 trp1- 2 leu2-3-112 his3-11 ade2-1 can1-100 GAL-A	[24]
W303-GAL-A-Cwc24	MATa/MATα ura3-52 trp1- 2 leu2-3-112 his3-11 ade2-1 can1-100 GAL-A-CWC24	[24]
TAP-Prp19	BY4741 MATa leu ura his TAP-Prp19	C. Guthrie
TAP-Brr2	BY4741 MATa leu; ura; his+; TAP-Brr2	C. Guthrie
TAP-Nip7	BY4741 MATa leu; ura; his+; TAP-Nip7	Euroscarf
WT	MATa; his3-1; leu2-0; lys2-0; ura3-0; CWC24	[24]
Δcwc24/GAL-CWC24	MATa; his3-1; leu2-0; lys2-0; ura3-0; CWC24::KANR, YCp111GAL-CWC24	[24]

### Splicing extracts, substrates and reactions

Splicing extracts were prepared from yeast cells using a ball mill (Retsch) for cell lysis following centrifugation at 15,000 rpm for 30 min and 37,000 rpm for 1 h 20 m. The extracts were dialyzed twice against 2 L buffer D (20 mM HEPES [pH 7.9], 0.2 mM EDTA, 50 mM KCl, 20% glycerol and 0.5 mM DTT) and frozen on liquid nitrogen. Cwc24p immunodepletion was carried out using protein A-sepharose beads (GE Healthcare) coupled to anti-Cwc24 antibody. One volume of splicing extract was diluted in buffer D and incubated with the same volume of beads. Wild type pre-U3 and pre-ACT1 were a gift from C. Guthrie (UC San Francisco, USA). The sequence between nucleotides 106–677 of TEF4 precursor was cloned into pGEM-T vector (Promega) under control of T7 promoter, generating pGEM-TEF4. Splice site and branchpoint mutations were performed using Quik-change site mutagenesis kit (Stratagene) and specific primers for 3′ splice site (Table 2), generating pre-U3 ACAC, and to the branchpoint site of pre-U3, generating pre-U3BP. Substrates were prepared by in vitro transcription using T7 RNA polymerase (Promega) after DNA linearization. Transcripts were treated with DNaseI (Fermentas), proteinase K (GE healthcare), phenol-chloroform extracted and precipitated with sodium acetate and ethanol. Splicing reactions were performed with 10 nM primary transcript, 60 mM potassium phosphate pH 7, 3.75 mM MgCl_2_, 2 mM ATP, 3% PEG 8000, 0.1 U RNAsin (Promega) and 120 µg splicing extract at 25°C for 30 min (wild type substrates) or 90 min (mutated substrates). Reactions were resolved by electrophoresis on a denaturing gel (urea 6 M/polyacrilamide 8%). After quantitation of the bands, a ratio between mature and precursor RNAs was calculated to determine the splicing efficiency in the reactions. Normalization was performed for each experiment, considering “mock” reactions as 100% efficient. Standard errors were calculated from biological replicates.

**Table 2:** Primers used in this study.

Primer	Sequence
snU1F	TCCTTGGTCACACACACATACG
snU1Rev	GGGAATGGAAACGTCAGCAA
snU2F	CGGCATCAAGAAACGGACTT
snU2Rev	GGGTCGCGACGTCTCTAACT
snU4F	GGATTCGTCCGAGATTGTGTTT
snU4Rev	CATGAGGAGACGGTCTGGTTTAT
snU5F	CGGGTGTTGTCTCCATAGAAACA
snU5Rev	AGGGCAGAAAAGTTCCAAAAAAT
snU6F	CGTGGACATTTGGTCAATTTGA
snU6Rev	TTGTAAAACGGTTCATCCTTATGC
ExU3 2.1	TCTATAGGAATCGTCACTCTTTGACTCT
Ex2snR17R	GACCAAGCTAATTTAGATTCAATTTCGG
EJ snR17F	GACGTACTTCATAGGATCATTTCTA
U3for132	TCAACCATTGCAGCAGCTTT
U3rev113	TCTGCTCCGAAATGAAAACTCTAGTA
TEF106F	TCTAGCGAGTTTGCTTCTTTGTTCCC
TEF677Rev	CCTTTGAAAGAAAGGAATGGACGAGC
pre-U3 mut sense	GACTAACACATTCTACACACGGATCATTTCTATAGGAATC
pre-U3 mut antisense	GATTCCTATAGAAATGATCCGTGTGTAGAATGTGTTAGTC
pre-U3mutBP sense	TTTAATTCAACCATTGCAGCAGCTTTTTACTAACACATTCTAC
pre-U3mutBP antisense	GTAGAATGTGTTAGTAAAAAGCTGCTGCAATGGTTGAATTAAA
EJ IWR1	TGTGCAGGCATTATTAATTGATGAG
IWR for117	GCGTTTGACTAACTAAGTTCATCTGG
IWR for166	GAAAAGAGTGAAGAAGCAGAAGTTCA
IWR rev294	ACAAAATGTCGATGATCTTCATGAG
EJ YRA1	AGGATGCTGTAAGAGAATTTTTTGC
YRA for981	GTGTATTGTCCCTTCCTTCTTTGATT
YRA for1062	CATCTCAAGTAGGTGGTGTTCAAAG
YRA rev1171	AGCCCTTCTGGCCAATTCA
U14 forward	CACGGTGATGAAAGACTG
Anti U14	CTCAGACATCCTAGGAAGG
SCR1 RevRT-PCR	GCACGGTGCGGAATAGAGA

### Immunoprecipitation and glycerol gradients

*S. cerevisiae* strains W303/GAL-A and W303/GAL-A-CWC24 were grown on minimal media with galactose. Cell extracts were prepared on buffer A containing 20 mM Tris-Cl [pH 8], 5 mM magnesium acetate, 150 mM potassium acetate, 1 mM DTT, 1 mM PMSF and incubated with IgG-sepharose (GE Healthcare) for 2 h at 4°C. Following three washes on the buffer A, beads were used for RNA extraction. For spliceosomes immunoprecipitations, splicing reactions were purified on linear glycerol gradients (10–30%) using a buffer containing 20 mM HEPES [pH 7.9], 50 mM KCl and 0.5 mM DTT. Gradients were run as described before [25] on SW65Ti rotor (Beckman) at 20,500 rpm for 16 h at 4°C. Sixteen 300 µl fractions were collected and cpm was measured on a cintilator counter (Perkin Elmer). Splicing complexes were immunoprecipitated using IgG-sepharose (GE Healthcare) for 3 h at 4°C following 3 washes on SCB-H buffer (150 mM KCl, 5 mM EDTA, 20 mM HEPES pH 7.9) [26].

### RT-qPCR and northern blot

Total RNAs from WT and *Δcwc24/GAL-CWC24* strains, and RNA from the beads were extracted [27] and used for cDNAs synthesis using random primers (Invitrogen). cDNAs were used for RT-qPCR reactions on a 7500 Real Time PCR (Applied Biosystems) using SYBR Green master mix (Fermentas). To determine the amount of RNAs in the samples, specific primers were used (Table 2). The comparative Ct method was used for the quantitation using mock-depleted reactions as a control. Differences between quantitation were tested using Student's T test and randomization T tests [28]. Total RNA was separated on 1.5% agarose or 8% acrylamide/6 M urea gels to resolve snRNAs. RNA was transferred to nylon membranes (Hybond-N+, GE Healthcare). These membranes were hybridized against ^32^P labeled probes specific for snRNAs U1, U2, U4, U5, U6 and for scR1 as a control [29].

### Expression and purification of recombinant Cwc24

GST-Cwc24 and His-Cwc24 were expressed in *E. coli* BL21 using pGEX-CWC24 [24] and pET28-CWC24, respectively. Expression was induced by addition of 0.5 mM IPTG to a mid-logarithmic phase culture at 37°C for 4 h. GST-Cwc24 extracts were prepared on a buffer containing 50 mM Tris [pH 8], 200 mM KCl, 1 mM DTT and 1 mM PMSF and incubated with GST-sepharose (GE Healthcare) for 1 h at 4°C. Following 5 washes in the same buffer as used for extract preparation, GST-Cwc24 was eluted by three incubations with 20 mM reduced glutathione on 50 mM Tris [pH 8]. GST-Cwc24 was loaded onto a heparin column (GE Healthcare) using buffer 50 mM Tris [pH 8], 100 mM KCl, 0.5 mM DTT. The protein was eluted over a linear 100 mM–1 M KCl gradient. His-Cwc24 extracts were prepared on a buffer containing 50 mM phosphate buffer [pH 7.2], 100 mM NaCl, 5% glycerol and loaded onto a Hi-Trap Chelating Ni (GE Healthcare). Elution was performed on a 50–250 mM imidazol gradient. His-Cwc24 was further cleaned on a SP-sepharose column, using a gradient 100 mM–1 M NaCl to elution.

#### Protein pull-down

*S. cerevisiae* strains TAP-Brr2 and TAP-Nip7 were grown on YPD. Cell extracts were prepared on buffer AGK (10 mM HEPES [pH 7.9], 1.5 mM MgCl_2_, 200 mM KCl, 10% glicerol, 0.5 mM DTT) with 1 mM PMSF and incubated with IgG-sepharose (GE Healthcare) for 2 h at 4°C. Supernatant was collected, beads were washed 4 times with AGK+400 mM KCl and beads were incubated with His-Cwc24 for 2 h at 4°C. Supernatant was collected, beads were washed 3 times with AGK and loaded on SDS-PAGE to perform western blot using anti-his antibody (Sigma).

## Results

### Cwc24p is a general splicing factor

**Figure 1:**
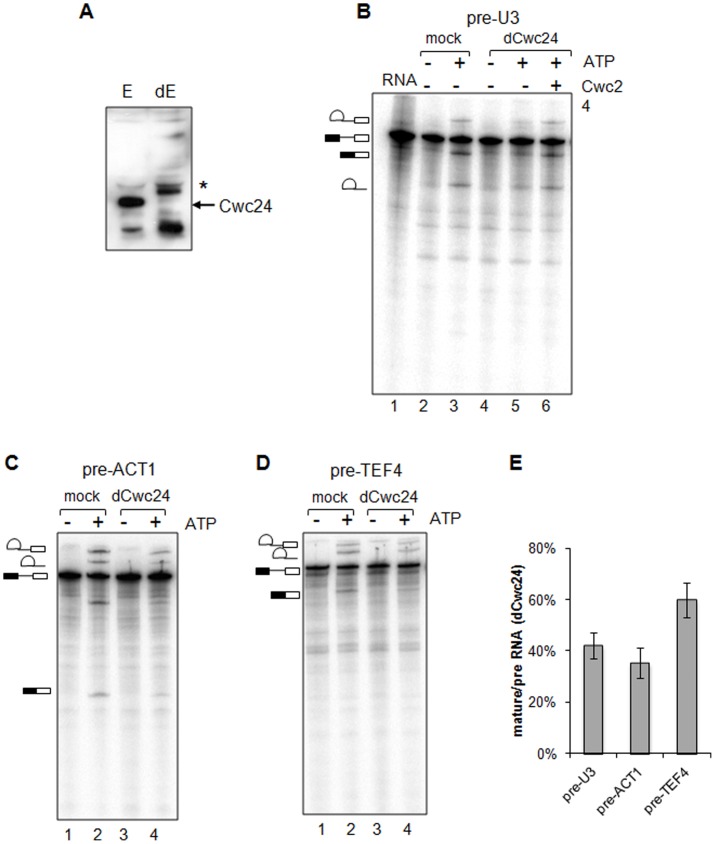
Immunodepletion of Cwc24p inhibits *in vitro* splicing. (**A**) Western blot using anti-Cwc24 to confirm immunodepletion of Cwc24p in splicing extracts. E, splicing extract, dE, depleted extract. The asterisk indicates an antiserum-raised band in the depleted extract. (**B**) *In vitro* pre-U3A splicing using mock- and Cwc24-depleted (dCwc24) extracts. Lanes 2 and 3 represent mock-depleted reactions, with or without ATP, as indicated. Lanes 4 to 6 represent reactions with Cwc24-depleted extracts, with or without ATP. Lane 6, addition of 1 µM recombinant GST-Cwc24 restores splicing. RNA, pre-U3A used in the reactions. (**C, D**) *In vitro* splicing of pre-ACT1 and of pre-TEF4. Addition of ATP to reactions is indicated, with mock- or Cwc24- depleted extracts as described in **B**. Intermediates are shown on the left, namely, primary transcript, intron-3′exon, intron-lariat, and mature RNA. (**E**) Quantitation of splicing efficiency of pre-U3, pre-ACT1, or pre-TEF4 substrates with dCwc24 extracts. Efficiency of splicing was calculated relative to mock reactions for 2 to 3 independent experiments. Standard error bars are shown on the graph. We used quantitations of bands corresponding to mature RNA and precursor RNA to calculate relative splicing efficiency (see Methods for further information).

Cwc24p was identified as a component of a multi-protein complex in association with Cef1p [21]. Previous studies using splicing microarray analysis revealed that the depletion of Cwc24p resulted in strong splicing defects for pre-snR17A and pre-snR17B transcripts (pre-U3 snoRNA), with milder effects on other primary transcripts [24]. The strong effect of Cwc24p depletion on pre-U3 snoRNA splicing was further confirmed by northern hybridization and primer extension analyses [24]. In order to investigate the mechanism by which Cwc24p affects pre-U3 splicing, we performed *in vitro* splicing reactions using extracts from wild-type yeast cells, which were immunodepleted of Cwc24p (Fig. 1). Immunodepletion was performed with anti-Cwc24 coupled protein A-sepharose beads, and confirmed by western blot (Fig. 1A). By comparing with mock-depleted extracts, a strong decrease in splicing efficiency of a synthetic pre-U3 snoRNA transcript was observed upon Cwc24p depletion (42.1%+/−5.1% on average, see Fig. 1B lane 5). This result was reproduced with different splicing extract preparations. Addition of 1 µM recombinant GST-Cwc24p to these reactions restored splicing (82.9% of splicing comparing with mock reactions, compare lanes 3 and 6, Fig. 1B), confirming Cwc24p as an important factor for pre-U3 splicing.

Previous analysis of the effect of Cwc24p on splicing was performed by using the conditional strain *Δcwc24/GAL-CWC24* grown in glucose medium for up to 8 hours, conditions in which Cwc24p could still be detected by western blot [24]. In those assays, splicing of RNAs other than pre-U3 was only slightly affected. In the assays performed here, on the other hand, Cwc24p could not be detected by western blot after the immunodepletion (Fig. 1A). In order to determine whether Cwc24p also affects the splicing of other primary transcripts *in vitro*, Cwc24p-immunodepleted extracts were used to test splicing with the pre-mRNAs pre-ACT1 and pre-TEF4. *TEF4* codes for a translation elongation factor and hosts a box C/D snoRNA (snR38) in its intron. Both TEF4 and ACT1 pre-mRNAs have similar lengths (∼1.5 kb) and similar intron features, as well as consensus sequences in 5′ and 3′ splicing sites, and branchpoint sequence. The absence of Cwc24p inhibited splicing of both pre-mRNAs, although to different extents (Fig. 1C, D). In comparison with mock-depleted reactions, Cwc24p-depleted reactions using pre-ACT1 showed reduction to 35.2% of splicing on average, and pre-TEF4 to 59.7% of splicing on average. Interestingly, both U3 and snR38 (found in TEF4 intron) are box C/D snoRNAs and were strongly affected upon Cwc24p depletion, which is consistent with previous *in vivo* data [24]. Also in agreement with previous *in vivo* observations, splicing of pre-TEF4 was affected by the absence of Cwc24p [24]. Although the overall effect of the depletion of Cwc24p on splicing was consistent for the *in vitro* and *in vivo* assays, differences in the extent of this effect were observed. It is possible that pre-ACT1 *in vivo* splicing was not as affected due to the short period of Cwc24p depletion [24]. Taken together, the splicing inhibition of different transcripts caused by the depletion of Cwc24p *in vitro* suggests a broader role for Cwc24p in splicing.

**Figure 2:**
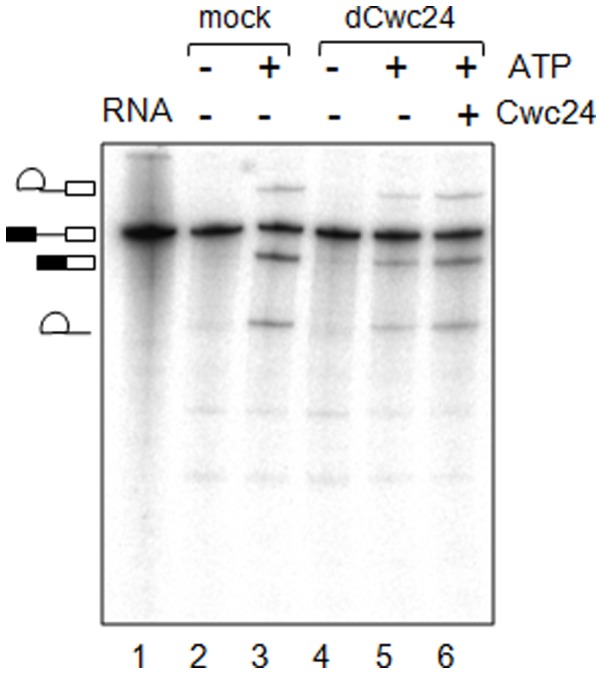
Immunodepletion of Cwc24p affects splicing of pre-U3BP. *In vitro* splicing reactions of pre-U3BP substrate, performed with mock- or Cwc24-depleted extracts, with or without ATP. A mutation on pre-U3 branchpoint site creates a consensus site (pre-U3BP) and splicing is less affected by the depletion of Cwc24p (lane 5), if compared with wild-type pre-U3A. Intermediates are shown on the left, namely, primary transcript, intron-3′exon, intron-lariat, and mature RNA. Efficiency of splicing was calculated relative to mock reactions for 3 independent experiments. Quantitation of bands corresponding to mature RNA and precursor RNA were used to calculate relative splicing efficiency. The average splicing efficiency of reactions performed with dCwc24 extracts was 46.4% (+/−7.1%). Addition of 1 µM GST-Cwc24 restores splicing on pre-U3BP.

In order to investigate the mechanism responsible for the effect of Cwc24p on pre-U3A splicing, we analyzed the features of this transcript. Apart from the fact that U3 is a box C/D snoRNA, other specific intronic features could impair optimal splicing and might explain the requirement of Cwc24p for splicing of this transcript. In addition to a short intron (157 nt), pre-U3A has a non-consensus branchpoint site (GACUAAC in pre-U3; whereas the consensus sequence is UACUAAC). It is therefore possible that the pairing between pre-U3 and snRNA U2, as well as the interactions within this region are less stable. In order to investigate the requirement of Cwc24p for the splicing of pre-U3, the non-consensus pre-U3A branchpoint was mutated to a consensus site, generating pre-U3BP. *In vitro* reactions using mock- and Cwc24p-depleted extracts showed that depletion of Cwc24p reduces splicing efficiency on this substrate (down to 46.4% on average, Fig. 2, lane 5). Addition of recombinant Cwc24p to the depleted reactions completely recovered the splicing efficiency in these reactions. Importantly, it should be noted that the non-consensus branchpoint site by itself would not account for the splicing deficiency observed for pre-U3, since the depletion of Cwc24p also caused inhibition of splicing of pre-TEF4, pre-ACT1, and pre-U3BP. These results suggest that Cwc24p is important for splicing in general, but given the strong effect of Cwc24p depletion on pre-U3, it is possible that this protein also facilitates the splicing of transcripts with non-consensus branchpoint sites.

**Figure 3:**
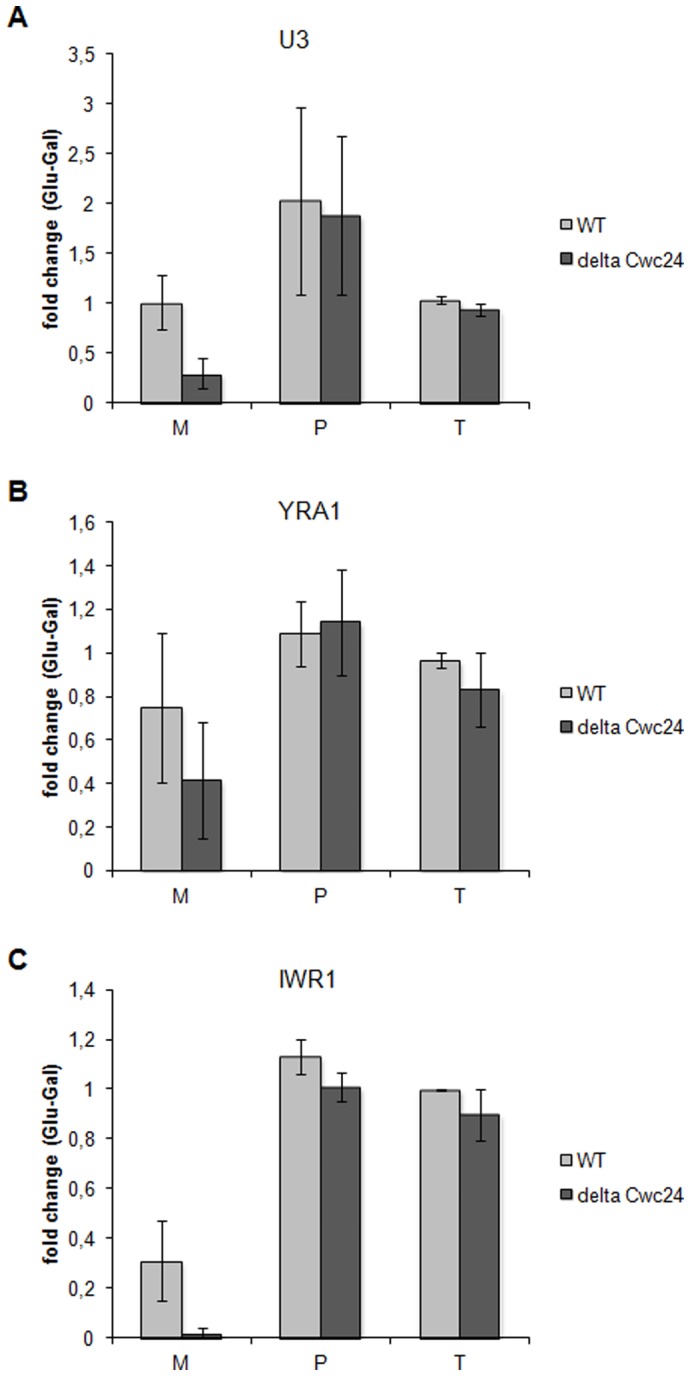
RT-qPCR reactions using RNAs from WT and *Δcwc24/GAL-CWC24* strains grown on galactose and shifted to glucose for 48 h. Reactions were performed with primers for mature (M), precursor (P) and total (T) RNAs. Three independent biological replicates were used and reactions were run in duplicates for technical replicates. The graphs show the average fold change values for each primer after shift from galactose to glucose. Standard errors were calculated on the basis of fold change variations between replicates. (**A**) Reaction with U3 primers. (**B**) Reaction with YRA1 primers. (**C**) Reaction with IWR1 primers. Samples were normalized using primers for snoRNA U14. A schematic representation of the primers complementary to different mRNAs regions is shown in Fig. S1.

To determine whether Cwc24p could facilitate splicing of other substrates with non-canonical branchpoints, we analyzed the effects of Cwc24p depletion on *in vivo* splicing of two other yeast transcripts, IWR1 and YRA1. Similar to pre-U3, these transcripts have branchpoint sites with a G instead of U in the first position. In addition to that, these transcripts have different intron features that could interfere with splicing progression, for example, YRA1 intron is unusually long for a yeast gene (766 nucleotides). IWR1 intron, on the other hand, is only 61 nucleotides long. To analyze the possible involvement of Cwc24p in the splicing of these mRNAs, we used the conditional strain *Δcwc24/GAL-CWC24* [24] grown in glucose for 48 h to completely deplete Cwc24p. Total RNA isolated from the cells growing either in galactose or in glucose medium was analyzed by RT-qPCR using specific primers for mature, precursor, and total RNAs (Fig. S1). In these experiments, three independent biological samples with two duplicates each were analyzed and the average fold change between glucose and galactose is shown (Fig. 3). The results showed that, similar to mature U3, mature IWR1 and YRA1 were reduced upon Cwc24p depletion *in vivo* (Fig. 3). Average mature U3 levels were reduced to 0.29, compared to the levels observed in galactose (randomization T test, P<0.05; [28]; Fig. 3A). The levels of mature YRA1 were reduced to 0.41 upon Cwc24p depletion *in vivo* (randomization T test, P<0.01; Fig. 3B). In the case of mature IWR1, average levels dropped to 0.02 upon Cwc24p depletion (randomization T test, P<0.01; Fig. 3C). Importantly, no significant difference in total RNA levels was observed between *Δcwc24/GAL-CWC24* cells grown in galactose or in glucose medium (randomization T test, P>0.2 in all comparisons), although IWR1 mRNA levels decreased when cells were transferred to glucose medium. The lower levels of IWR1 mRNA, however, are not dependent on Cwc24p, since it was observed in both strains. Quantitative PCR data was normalized with snoRNA U14, whose levels are independent of splicing. These results showed that the *in vivo* depletion of Cwc24p affects splicing of primary transcripts containing non-consensus branchpoint sequences, confirming the essential role of Cwc24p for splicing *in vivo* and *in vitro*.

### Cwc24p associates with the spliceosome

**Figure 4:**
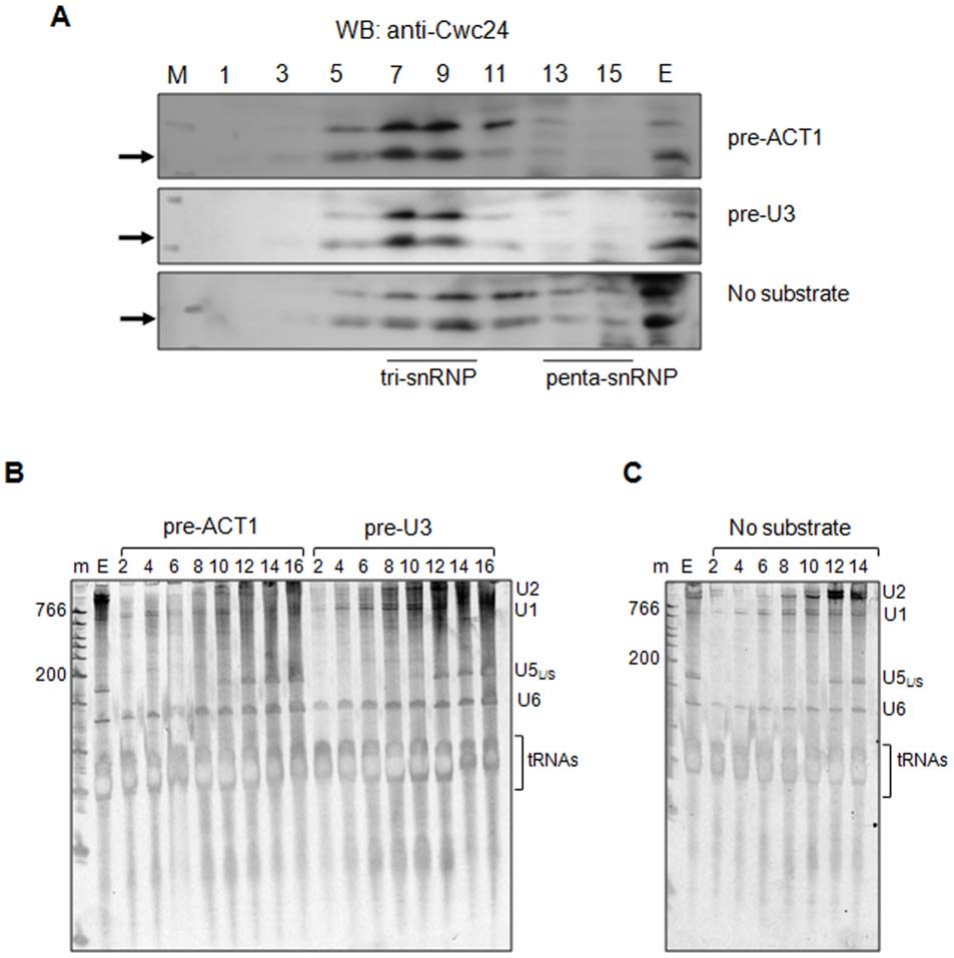
Cwc24p association with splicing complexes. (**A**) Anti-Cwc24 western blot after linear glycerol gradient fractionation. Splicing reactions were prepared with pre-ACT1, pre-U3, or without substrate. Odd-numbered fractions from top to bottom of the gradient are shown. E, splicing extract. (**B**, **C**) Even-numbered fractions were used for analysis of snRNAs after electrophoresis on denaturing polyacrylamide gels and staining with SYBRgold (Invitrogen). The positions of RNA species are shown on the right. M, molecular mass marker; E, splicing extract.

In order to determine whether Cwc24p was associated with splicing sub-complexes, we performed centrifugation of *in vitro* reactions on 10–30% linear glycerol gradients. *In vitro* splicing reactions of pre-U3 and pre-ACT1 were separated by using glycerol gradients in 50 mM KCl buffer, conditions that allow for the separation of different splicing subcomplexes [30]. On these gradients, Cwc24p sedimented in fractions 5–11, with a peak in fractions 7–9 independently of the substrate used in the reaction (Fig. 4A; upper and middle panels). Fractions 7–9 correspond to sedimentation of tri-snRNPs [30]. Analysis of the Cwc24p sedimentation on gradients of splicing extracts with no substrate and no ATP added to the reaction showed that the protein is distributed from fraction 5 through 15, suggesting a less stable association of Cwc24p with splicing subcomplexes when no substrate is added to the reaction (Fig. 4A; lower panel).

The profile of snRNAs sedimentation in these gradients was also analyzed. snRNAs were detected in fractions 6–16, which includes tri- and penta-snRNPs complexes [Fig. 4B, C, 30]. In the presence of either pre-U3 or pre-ACT1 substrates and ATP, however, U2 snRNA is concentrated in fractions 6–12, whereas in their absence, U2 is mainly found in fractions 8–14 (Fig. 4B, C). The sedimentation of Cwc24p upon ATP addition and under splicing conditions is similar to that of the snRNAs in these gradients and suggests that Cwc24p might be associated with larger complexes, such as splicing sub-complexes.

**Figure 5:**
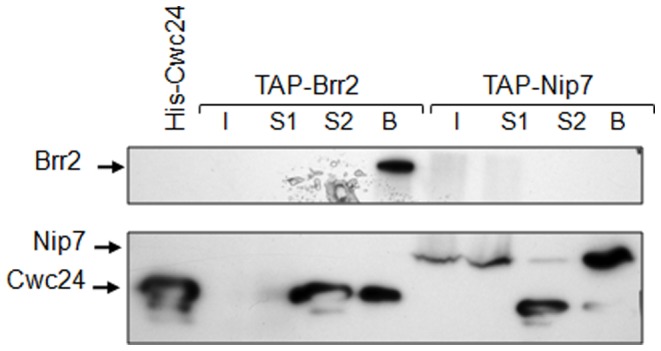
Interaction between Brr2p and Cwc24p. A pull-down analysis was performed using *S. cerevisiae* TAP-Brr2p and TAP-Nip7p (as a control) and *E. coli*-expressed His-Cwc24p. TAP-Brr2 and TAP-Nip7 extracts (I) were bound to IgG beads, supernatant was collected (S1) and His-Cwc24 was added to the beads. After that, supernatant was collected (S2) and beads were loaded on the gel. Western blot was performed using anti-his antibody. Proteins are represented on the left, Brr2p (246 kD), Cwc24p (30 kD) and Nip7p (21 kD).

Cwc24p was isolated in a multi-protein complex interacting with the NTC component Cef1p [16], and showed to interact directly with Cef1p and Nop17p, a box C/D snoRNP assembly factor [24], [27]. In order to investigate other spliceosome proteins that might interact with Cwc24p, we performed the yeast two-hybrid assay with Cwc2p, Yju2p, Nam8p, Mud1p and Brr2p. This analysis identified the Cwc24p interaction with Brr2p (data not shown). The ATP dependent RNA-helicase Brr2p is associated with U5 snRNP and promotes U4/U6 dissociation, an important rearrangement for catalytic activation [31]. Pull-down assays using *S. cerevisiae*-expressed TAP-Brr2p and *E. coli*-expressed His-Cwc24p followed by a western blot using anti-his antibody confirmed that the interaction is specific. In these assays, TAP-Brr2p was immobilized with IgG-sepharose beads, followed by incubation with recombinant His-Cwc24p, which was efficiently retained in the bound fraction (Fig. 5). Nip7p is a nuclear protein involved in pre-rRNA processing [32] and the fusion TAP-Nip7p was used as a control in this experiment, confirming that the His-Cwc24p-TAP-Brr2p interaction is specific (Fig. 5).

**Figure 6:**
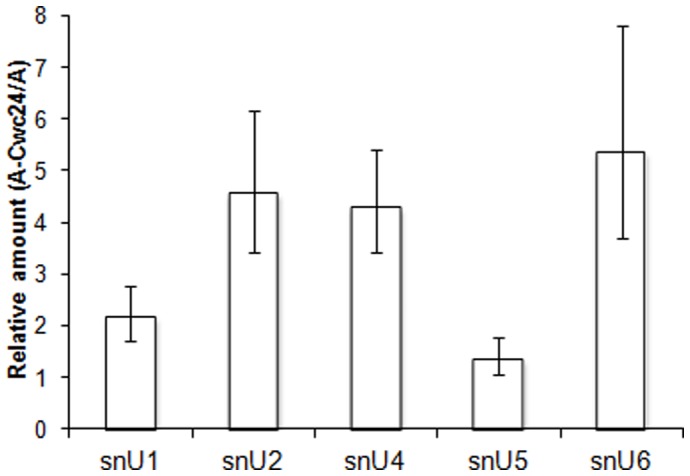
ProtA-Cwc24p co-immunoprecipitates snRNAs. RT-qPCR analysis of co-IP samples from ProtA and ProtA-Cwc24p using specific primers for the snRNAs. Relative amounts of co-immunoprecipitated RNA were calculated by comparing A-Cwc24p with ProtA. To quantify the results, the ΔΔCt method was used and the y-axis shows the ratio between ProtA-Cwc24 and ProtA. Standard error bars were calculated from the results of triplicates.

In addition to its interaction with Cef1p and Brr2p, it is possible that Cwc24p interacts with other snRNP components. In order to identify the snRNPs to which Cwc24p could bind, we performed a snRNPs co-immunoprecipitation using ProtA-Cwc24p or ProtA as a control. The co-immunoprecipitated RNAs were extracted from the IgG-sepharose beads, used for cDNA synthesis with random primers, and subjected to qPCR using specific primers for each snRNA. The results of three independent experiments showed that ProtA-Cwc24p significantly co-precipitated the snRNAs U2 and U6 (Student's T test, P<0.05; Fig. 6). Interestingly, ProtA-Cwc24p also precipitated U4 snRNA (Fig. 6). NTC factors have been shown to have different affinities for individual snRNPs. Cwc2p, for instance, binds U2 and U6, whereas Cef1p associates with U2, U5, and U6, and Bud31p co-immunoprecipitates U5 and U6 [21], [33], [34]. As shown here, Cwc24p, also a factor interacting with the NTC complex, co-immunoprecipitates U2, U6 and U4. These results suggest the early Cwc24p association with the spliceosome, before its activation takes place. The direct Cwc24p interactions with Cef1p and Brr2p, a U5 snRNP component, and the stronger precipitation of U2 and U6, strengthen the hypothesis of Cwc24p being associated with the spliceosome core.

To determine whether Cwc24p is stably associated with one of the splicing intermediates through its interaction with the snRNPs, spliceosomes were *in vitro* assembled using pre-U3A in wild-type splicing extracts. These reactions were immunoprecipitated with either mock or anti-Cwc24 coupled beads. The splicing substrate, intermediates and products, were analyzed by RT-qPCR and visualized on a denaturing gel (Fig. S2). These analyses showed that Cwc24p is neither bound to precursor U3, nor to the intron-lariat intermediate. Cwc24p association with splicing sub-complexes is probably due to its direct interaction with splicing factors. Consistently, proteomic analysis detected Cwc24p enrichment in B^act^ [3].

### Cwc24p is important for Prp19p association with the spliceosome

**Figure 7:**
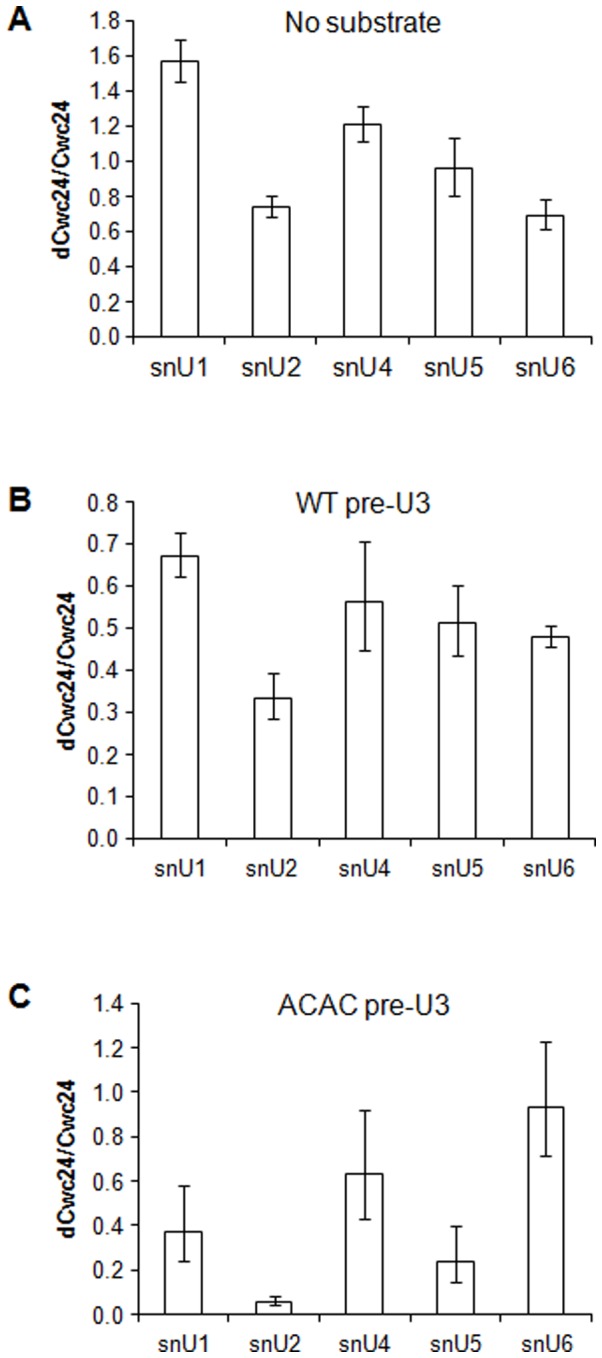
Immunoprecipitation of splicing complexes with TAP-Prp19p is affected by the presence of Cwc24p. TAP-Prp19p mock- or Cwc24-depleted yeast splicing extracts were used in these reactions. After immunoprecipitation with IgG-sepharose beads, RNAs were extracted from the beads and cDNAs were synthesized and used as templates for qPCR reactions. To quantify the results, the ΔΔCt method was used and the y-axis shows the ratio between Cwc24-depleted (dCwc24) and mock (Cwc24) reactions. (**A**) Reactions performed using mock- and Cwc24- depleted extracts, without substrate. (**B**) Reactions with wild-type pre-U3. (**C**) Reactions with pre-U3 ACAC. Samples of reactions with substrate were normalized using a primer that hybridizes to the 3′ exon on pre-U3. By using this control reaction, we normalized the Ct values by the amount of precursor RNA, which is 10 nM for all reactions, independently of the reaction efficiency. Samples from the extracts with no substrate were normalized using a primer to snoRNA U14. Standard error bars were calculated from the results of triplicates.

Since Cwc24p was isolated as a Cef1-interacting protein and Cef1p is part of the Prp19-complex (NTC) [21], we next addressed the question of whether Prp19p binding to the spliceosome was influenced by Cwc24p. We therefore performed co-immunoprecipitation of splicing complexes with TAP-Prp19p in extracts depleted of Cwc24p. First, we analyzed the snRNA levels on mock- and Cwc24-immunodepleted extracts using RT-qPCR. We observed that TAP-Prp19 extracts depleted of Cwc24p showed a slight reduction in the levels of U2 and U6 snRNAs (Fig. 7A), compared to mock-depleted extracts. These results suggest that Cwc24p affects Prp19p association with splicing complexes.

To analyze the possible effects of Cwc24p depletion on the snRNAs levels *in vivo*, a northern hybridization was performed using RNAs from WT and *Δcwc24/GAL-CWC24* strains grown in galactose or in glucose. The results show that the levels of the snRNAs are not significantly affected by the depletion of Cwc24p (Fig. S3).

Based on the effect of the depletion of Cwc24p on Prp19p binding to snRNPs U2 and U6, we next checked its effect on splicing assembly. Splicing extracts prepared from TAP-PRP19 strain were immunodepleted of Cwc24p and used to assemble spliceosomes on pre-U3 snoRNA. After assembly, the reactions were subjected to 10–30% linear glycerol gradients, and fractions 9–12 (corresponding to tri- and penta-snRNPs) were subsequently subjected to affinity columns to purify TAP-Prp19-associated complexes. RNA was extracted from the bound material and used in RT-qPCR reactions with primers for the snRNAs. The results showed that, upon depletion of Cwc24p, the amount of U2 snRNA precipitated by TAP-Prp19p under splicing conditions is strongly reduced, down to 0.33 compared with the mock-depleted extracts (Fig. 7B). Depletion of Cwc24p also caused a decrease in the amounts of U6 and U5 bound to TAP-Prp19-complexes (0.47 and 0.51 of the levels observed on the mock-depleted extract, respectively; Fig. 7B), but the effect was not as strong as that observed for U2. The reduction in the levels of these snRNAs co-precipitating with Prp19p after Cwc24p depletion indicated that the spliceosome was not correctly assembled. During spliceosome assembly, the presence of Prp19p and the NTC is important for promoting the rearrangements required for the catalytic activation [17]. The data shown here strongly suggest that Cwc24p has an early role stabilizing U2 snRNA and helping the spliceosome perform the rearrangements needed for complex activation. In the absence of Cwc24p, therefore, these rearrangements are impaired probably inhibiting assembly as well.

The reduced amounts of U2, U5 and U6 snRNAs coimmunoprecipitated with TAP-Prp19p upon Cwc24p depletion are consistent with the precipitation of these snRNAs with ProtA-Cwc24p (see Fig. 6). In order to investigate whether the defect on assembly would interfere with either first or second step splicing reactions, a similar experiment with TAP-Prp19p mock- and Cwc24-immunodepleted extracts was performed, using a substrate in which the 3′ splice site was mutated from ACAG to ACAC (ACAC pre-U3). Although the first step of splicing can occur normally in this substrate, the second step is blocked and thus complex assembly stalls after the first catalytic reaction. In this case, depletion of Cwc24p caused a greater reduction on U2 and U5 snRNAs levels (down to 0.05 and 0.20 compared to the mock levels, respectively) precipitated with TAP-Prp19p. However, the same reduction was not observed for U6 snRNA (Fig. 7C). This result suggests that Cwc24p is associated with the complex before the first step of catalysis, but upon Cwc24p depletion, U2 snRNA is poorly associated and thus probably not stabilized into this complex. However, U6 snRNA levels showed that Cwc24p does not directly affect its association or stabilization. In fact, on the mutant ACAC substrate and under Cwc24p depletion, U6 levels were near normal. These results further indicate that Cwc24p is mainly involved in the stabilization of the U2 association with the spliceosome.

## Discussion

Although many spliceosomal components are known, the interaction dynamics between them and the role the sub-complexes play in primary transcripts splicing regulation remain to be determined [1], [3]. For the yeast *S. cerevisiae*, it is known that interactions between snRNAs and specific proteins can enhance or repress splicing activity and/or regulate spliceosome activation under different environmental conditions or developmental stages [35], [36]. It is possible that specific components and its interactions with the primary transcripts facilitate and control this process. We have previously shown that the depletion of Cwc24p affects splicing of pre-U3 snoRNA *in vivo* [24]. However, one of the greatest challenges that emerged from that work was to understand whether Cwc24p associates and interferes with pre-U3 spliceosomes. In this work, we performed *in vitro* splicing experiments with Cwc24-immunodepleted extracts, using different substrates. Our data showed that this 30 kDa protein is a general splicing factor required for the stable association of U2 snRNP with pre-RNAs and thus important for spliceosome assembly, especially of those primary transcripts containing non-canonical branchpoint sequences.

In accordance with the hypothesis of Cwc24p being a general splicing factor, depletion of Cwc24p in *in vitro* splicing reactions led to a reduction of the mature RNAs formed from the three different substrates used, pre-U3, pre-TEF4, and pre-ACT1. Splicing of all three transcripts tested was inhibited upon depletion of Cwc24p. Consistently with previous microarray data, U3 was very strongly affected. Interestingly, both U3 and snR38 (the snoRNA found in TEF4 intron) are box C/D snoRNAs. This might indicate a connection between the splicing and box C/D snoRNP assembly machineries. The great majority of mammalian snoRNAs are coded in introns and, consistently, snoRNP assembly is dependent on interactions between components of the spliceosome and the snoRNP machineries. IBP160 was isolated as one of such proteins, connecting splicing of primary transcripts containing snoRNAs in the introns to proteins of snoRNP complex [37], [38]. However, in *S. cerevisiae*, only 7 snoRNAs are coded within introns, 6 of which are snoRNAs of box C/D [39]. The splicing defects observed upon Cwc24p depletion along with its interactions with components of splicing machinery (Cef1p and Brr2p) and snoRNP assembly (Nop17p) [24], could indicate that this protein mediates these processes. In accordance with that, the addition of recombinant Cwc24p restored the efficiency of splicing of pre-U3 snoRNA, confirming that the splicing defect is specifically due to the depletion of Cwc24p. Taken together, the splicing defects observed under Cwc24p depletion indicate this is a general splicing factor, but especially required for pre-U3 splicing.

In *S. cerevisiae*, the U3 snoRNA is transcribed from two different genes, snR17A and B, which code for pre-snoRNAs containing 157-nt or 130-nt introns, respectively. Despite its canonical 5′ and 3′ splice sites, the branchpoint sequence is not conserved in this pre-snoRNA, with a G instead of a U in the first position. The mature U3 snoRNA is essential for the initial cleavages of the ribosomal RNA precursor [40] and, consistently, depletion of Cwc24p also affects pre-rRNA processing [24]. In fact, our data supports the hypothesis that splicing of this transcript, that has a non-consensus branchpoint sequence, depends on Cwc24p. Yeast splicing is strictly regulated and consensus sequences are essential to guide the spliceosome [4], through interactions with its components and with the precursor RNA. In *S. cerevisiae*, in addition to pre-U3 snoRNA (snR17A and snR17B), twelve other transcripts have this non-canonical branchpoint site (Ares Lab Yeast Intron Database, http://intron.ucsc.edu/yeast4.1/). We also tested *in vivo* splicing on two such transcripts, IWR1 and YRA1, and showed that Cwc24p depletion severely affected splicing of both. These results confirm the hypothesis of an important role for Cwc24p on splicing of primary transcripts with non-canonical branchpoint sites.

During spliceosome assembly, the branchpoint site on the precursor RNA is important to recruit and stabilize the U2 snRNP. Several rearrangements on the U2 snRNP particle and its pairing with U6 snRNA are important for catalytic activation and their positioning on the primary transcript [6], [8], [41]. U2 snRNA pairing with the branchpoint site also prevents the reaction from happening until rearrangements promoted by Prp2p allow the transesterification reaction to occur [10]. In this sense, a non-consensus branchpoint might be weakly bound by U2 snRNP, destabilizing the complex and leading to inefficient spliceosome activation. Indeed, our data shows that Cwc24p depletion causes a strong reduction on U2 and U6 association with pre-U3A spliceosomes, which could be due to the fact that either U2 or the pair U2/U6 is unstable in these complexes. Interestingly, Cwc24-depleted reactions using a 3′ splice site mutant substrate (ACAC pre-U3), which blocks spliceosome progression after the first step of splicing, caused a reduction in U2 levels but did not alter U6 levels bound to the complexes. This suggested that the depletion of Cwc24p destabilized the association of U2 snRNA, but did not affect the U6 snRNA recruitment. It is possible that Cwc24p facilitates either recruitment or stabilization of U2 snRNP on the pre-U3 substrate. However, our data showing the importance of Cwc24p for snRNAs precipitation with TAP-Prp19 spliceosomes, along with previous data showing the enrichment of Cwc24p on B^act^ complex [3], and its association with NTC complex [21] favor the latter hypothesis. Therefore, Cwc24p may be important for the stabilization of U2 snRNP, helping to promote the rearrangements necessary for the catalytic activation.

This suggests an important role for Cwc24p on spliceosome assembly, which ultimately leads to efficient splicing. It is also important to consider that U2 association and stabilization on the spliceosome is highly dependent on U2 snRNP proteins, for example the SF3a/b proteins [10], [42], [43]. Although we were unable to detect an interaction between these proteins and Cwc24p, it is possible that transient interactions occur. Interestingly, recent high-throughput analysis with the human orthologue of Cwc24p (RNF113) showed that this protein interacts with subunits of the B^act^ complex in the yeast two-hybrid system [44]. Weak splice sites have been shown to be subjected to specific regulation by protein components of the spliceosome. Hub1p facilitate splicing of transcripts with a deficient 5′ splice site [15]. The association of MER2 transcript weak 5′ splice site with U1, for example, is dependent on the interaction between Mer1p and U1 snRNP proteins during meiosis [45]. Mer1p binds to an intronic enhancer and mediates U1 snRNP association leading to spliceosome assembly on this substrate [45], [46], [47].

The spliceosome is a multi-megadalton machinery composed of ∼60 protein components but more than 100 proteins participate in its assembly [1], [48]. Cwc24p was isolated on a multi-protein complex in association with Cef1p [21]. Proteomic analysis showed that, similarly to Prp19p and Cef1p, Cwc24p is already present in B complex, with enrichment in B^act^ complex just prior to activation. But, also similarly to most of other Cwc proteins, fewer Cwc24p peptides were detected in C complex, indicating that its association with the catalytically active complex is less stable [3], [49]. Prp19p and Cef1p are major subunits of the NTC complex, and along with other components, promote several rearrangements important for catalytic activation [17]. Cwc24p physically interacts with Cef1p [21], [24], and our results showed that it is required for Prp19p association with the spliceosome. In the absence of Cwc24p, either because the NTC is incomplete, or because some essential interactions with other splicing factors are missing, the binding of Prp19p to the spliceosome is compromised. Regarding Cwc24p role in U2 binding to the primary transcripts, it is possible that Cwc24p acts as a regulatory protein that indicates when the spliceosome is ready to perform catalysis. In addition, interaction with Brr2p might be important for positioning and stabilizing Cwc24p on the catalytic core of the spliceosome. In this sense, other Cwc proteins have shown strong effects on splicing regulation. Cwc21p and Cwc25p are important for rearrangements prior to the first step reaction, regulating the spliceosome activation [19], [20].

In summary, our data shows that Cwc24p affects splicing in general by facilitating the stabilization of U2 on primary transcripts spliceosomes. This stabilization is especially important in the case of primary transcripts containing non-consensus branchpoint sequences. During the final preparation of this article a study was published on the Prp2-mediated proteins rearrangements at the catalytic core of the spliceosome [50]. In that study it is reported that Cwc24p is stably bound to B^act^ complex, but dissociates from it upon catalytic activation by Prp2p during transition to B*. That work complements the data reported here and confirms an early role for Cwc24p during splicing. In addition, Cwc21p, another protein associated with the NTC complex, was recently shown to be involved in the stabilization of the HRB1 pre-mRNA (which has a non-consensus branchpoint sequence) in the spliceosome catalytic center (Gautam, A., Grainger, R., Barrass, D. and Beggs, J.D.; personal communication), further suggesting the importance of the NTC complex for stabilizing the spliceosome on primary transcripts.

## Supporting Information

Figure S1 **Schematic representation of the transcripts regions complementary to the specific primers used for the RT-qPCR reactions shown in** **Figure 3** **.** Primers for the mature RNAs are complementary to the region spanning the exon junctions and 3′ exons; the precursor RNAs were detected using primers complementary to regions in the introns and 3′ exons; total RNAs were detected with forward and reverse primers for the 3′exons. EJ, primers for the exon junctions; 3′EF, forward primers for 3′ exons; 3′ER, reverse primers for the 3′ exons; IF, forward primers for introns.
(TIF)

Figure S2 **Immunoprecipitation of RNA intermediates with Cwc24p from** ***in vitro*** **reactions.** Reactions were carried out with wild-type splicing extracts using pre-U3A, then immunoprecipitated with anti-Cwc24 coupled Protein A-sepharose beads or mock-Protein A sepharose beads. RNAs were extracted from the input (I), flow-through (S) and pellet (P) samples. (**A**) RT-qPCR data using primers for mature U3 and intron-lariat intermediate (U3 for132 and U3 rev113), the graph shows the amount of RNA relative to the input sample. (**B**) Denaturing gel using input (I), supernatant (S) and pellet (P) samples of anti-Cwc24 and mock immunoprecipitations. Reaction intermediates are shown on the left, namely pre-U3 and mature U3.
(TIF)

Figure S3 **Analysis of the effect of Cwc24p depletion on snRNAs levels.** Total RNA was extracted from WT and *Δcwc24/GAL-CWC24* strains grown in galactose and shifted to glucose for 48 h. snRNAs U1, U4, U5 and U6 were resolved on acrylamide gel and snRNA U2 on an agarose gel. Northern blot was performed using specific probes for each snRNA.
(TIF)
