# Supplementary figures and images for: Cwc24p Is a General Saccharomyces cerevisiae Splicing Factor Required for the Stable U2 snRNP Binding to Primary Transcripts
Source: PLoS One. 2012 Sep 24;7(9):e45678. doi: 10.1371/journal.pone.0045678 (PMC3454408; doi:10.1371/journal.pone.0045678)

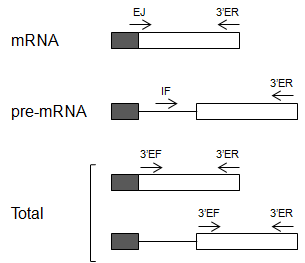

Supplement: Figure S1 — Schematic representation of the transcripts regions complementary to the specific primers used for the RT-qPCR reactions shown in Figure 3 . Primers for the mature RNAs are complementary to the region spanning the exon junctions and 3′ exons; the precursor RNAs were detected using primers complementary to regions in the introns and 3′ exons; total RNAs were detected with forward and reverse primers for the 3′exons. EJ, primers for the exon junctions; 3′EF, forward primers for 3′ exons; 3′ER, reverse primers for the 3′ exons; IF, forward primers for introns. (TIF) [file pone.0045678.s001.tif]

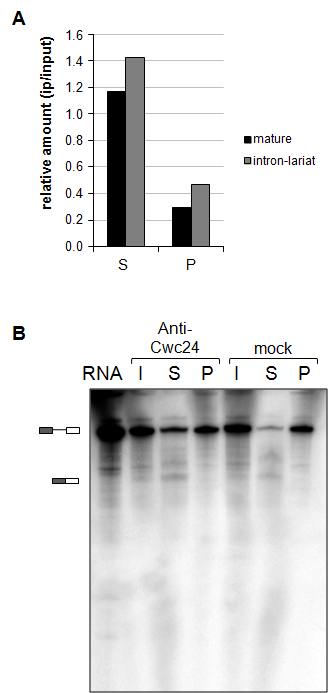

Supplement: Figure S2 — Immunoprecipitation of RNA intermediates with Cwc24p from in vitro reactions. Reactions were carried out with wild-type splicing extracts using pre-U3A, then immunoprecipitated with anti-Cwc24 coupled Protein A-sepharose beads or mock-Protein A sepharose beads. RNAs were extracted from the input (I), flow-through (S) and pellet (P) samples. (A) RT-qPCR data using primers for mature U3 and intron-lariat intermediate (U3 for132 and U3 rev113), the graph shows the amount of RNA relative to the input sample. (B) Denaturing gel using input (I), supernatant (S) and pellet (P) samples of anti-Cwc24 and mock immunoprecipitations. Reaction intermediates are shown on the left, namely pre-U3 and mature U3. (TIF) [file pone.0045678.s002.tif]

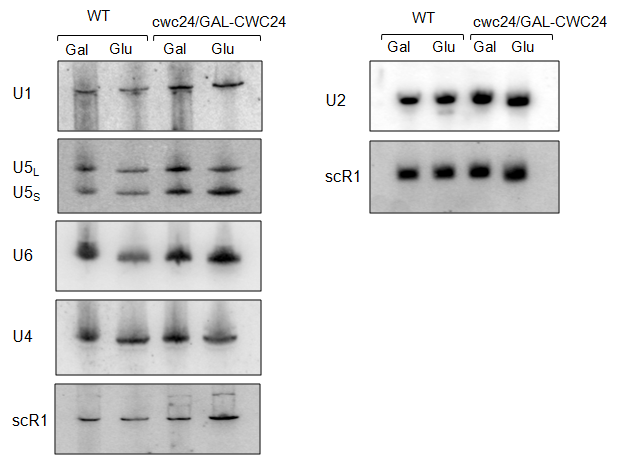

Supplement: Figure S3 — Analysis of the effect of Cwc24p depletion on snRNAs levels. Total RNA was extracted from WT and Δcwc24/GAL-CWC24 strains grown in galactose and shifted to glucose for 48 h. snRNAs U1, U4, U5 and U6 were resolved on acrylamide gel and snRNA U2 on an agarose gel. Northern blot was performed using specific probes for each snRNA. (TIF) [file pone.0045678.s003.tif]
